# Real-world persistence and compliance of denosumab versus alendronate among postmenopausal women with osteoporosis in Asia–Pacific

**DOI:** 10.1007/s00774-025-01663-2

**Published:** 2025-11-19

**Authors:** Tang Ching Lau, Dong-Gune Chang, Chung-Hwan Chen, Shuang Huang, Edith M. C. Lau, Sheung-Wai Law, Young-Kyun Lee, Cae Tolman, Laura Canals, See-Hwee Yeo, Jing Yu, Peter R. Ebeling

**Affiliations:** 1 Department of Medicine, Yong Loo Lin School of Medicine, National University of Singapore, Singapore, Singapore; 2 Department of Orthopedic Surgery, College of Medicine, Inje University Sanggye Paik Hospital, Inje University, Seoul, South Korea; 3 Department of Orthopedics and Orthopedic Research Center, College of Medicine, Regeneration Medicine and Cell Therapy Research Center, Kaohsiung Municipal Ta-Tung Hospital and Kaohsiung Medical University Hospital, Kaohsiung Medical University, Kaohsiung, Taiwan; 4 Amgen (Europe) GmbH, Rotkreuz, Switzerland; 5 Hong Kong Orthopaedic and Osteoporosis Center for Treatment and Research, Hong Kong, China; 6 Department of Orthopaedics and Traumatology, The Chinese University of Hong Kong, Hong Kong SAR, China; 7 Seoul National University Hospital, Seoul National University College of Medicine, Seoul, South Korea; 8 Amgen Australia, Sydney, Australia; 9 Department of Medicine, Amgen Europe, Amgen GmbH, Suurstoffi 22, 6343 Rotkreuz, Switzerland; 10 Real World Solutions, IQVIA Solutions Asia, Singapore, Singapore; 11 Real World Solutions, IQVIA Solutions Asia, Beijing, China; 12 Department of Medicine, School of Clinical Sciences at Monash Health, Monash University, Melbourne, Australia; 13 IQVIA Data Science Advanced Analytics, The Exchange Twin Tower (West), B-12 Jianguomenwai Avenue, Chaoyang, Beijing, China

**Keywords:** Osteoporosis, Denosumab, Alendronate, Medication persistence, Medication compliance

## Abstract

**Introduction:**

To compare 12-month real-world treatment persistence and compliance in postmenopausal women prescribed denosumab or alendronate for osteoporosis in Asia–Pacific.

**Materials and methods:**

This prospective cohort study enrolled women aged ≥ 50 years from Australia, Taiwan, South Korea, Hong Kong and Singapore between 2019–2021. Study participants were prescribed bi-annual denosumab injection or weekly alendronate, based on physicians’ decision. Medication persistence and compliance were estimated over 12 months of follow-up. Multivariable logistic regression was used to determine if treatment type (denosumab or alendronate) was significantly associated with persistence and compliance, while adjusting for age, fracture history, baseline BMD, prior osteoporosis treatment, and prior treatment with oral glucocorticoids. Adjusted odds ratios (aORs) and 95% confidence intervals (CIs) were calculated.

**Results:**

Among 686 enrolled women, 580 completed their 12-month visit (301 in denosumab and 279 in alendronate group). At baseline, those in the denosumab group were older and more likely to have received prior osteoporosis treatment. Overall treatment persistence and compliance were 72.4% and 70.7%, respectively. Patients receiving denosumab exhibited higher persistence (82.7% vs. 61.3%,* P* < 0.001) and compliance (86.0% vs. 54.1%, *P* < 0.001), versus those on alendronate. Regression results also showed that patients on denosumab were more likely to be persistent (aOR = 3.08; 95% CI 2.04–4.63) and compliant (aOR = 5.32; 95% CI 3.45–8.21) with treatment. Gastrointestinal disorders were more frequently reported in the alendronate group, while bone and joint injuries were more common in the denosumab group.

**Conclusion:**

Postmenopausal women with osteoporosis were more likely to be persistent and compliant with denosumab, compared with weekly oral alendronate.

**Supplementary Information:**

The online version contains supplementary material available at 10.1007/s00774-025-01663-2.

## Introduction

Osteoporosis is a chronic bone disease characterized by reduced bone mass and structure, which increases the risk of fractures, contributing to morbidity and mortality [1]. It is a major non-communicable disease and the most common bone disease that poses a significant burden with great economic costs [2]. A recent meta-analysis of cross-sectional and longitudinal studies revealed that the all-time global prevalence of osteoporosis was 19.7% [3]. Osteoporosis is predominantly observed in women, with age and menopause being significant risk factors. Postmenopausal elderly women are more likely to develop osteoporotic fractures than their male counterparts [4]. A systematic review conducted in 2022 reported that up to 30% of women aged 40 years and above, and up to 10% of men living in middle- and higher-income Asia–Pacific countries, were affected by osteoporosis [5]. It was projected that osteoporotic fractures would rise rapidly worldwide over the next few decades due to aging populations. By 2050, the global annual number of hip fractures is estimated to double (compared with 2018) [6], with 50% of them occurring in Asia [7].

Postmenopausal women at high fracture risk should generally be treated for osteoporosis [8]. Appropriate and timely treatment can reduce the risk of osteoporotic fractures and resultant physical disability, impaired quality of life, and even mortality. Bisphosphates such as alendronate are commonly recommended as first-line treatment for osteoporosis [9, 10]. They work by binding to actively remodeling bone surfaces and inhibiting bone remodeling [11]. A once-weekly dose of oral alendronate showed similar effectiveness as a daily dosage regimen [12], but it has to be taken continuously for several years. Long-term use of oral bisphosphonates may be associated with reduced medication persistence, potentially compromising treatment effectiveness [13]. Denosumab is a newer biologic agent used in the treatment of osteoporosis and is usually recommended as second-line therapy globally [9, 10]. It is a human monoclonal antibody that acts by inhibiting the *receptor activator of nuclear kappa-B ligand (RANKL)*, a key stimulator of osteoclast activity and mediator of bone resorption [14]. Denosumab offers the advantage of lower administration frequency compared with oral bisphosphonates, as it requires a subcutaneous injection only every 6 months [15]. Both denosumab and bisphosphonates have been shown to improve bone mineral density (BMD) and reduce the risk of fractures [16, 17]. At 12 months, denosumab showed superiority in BMD gains in total hip, femoral neck, trochanter, lumbar spine, and one-third radius in postmenopausal women with osteoporosis [18, 19]. In addition, studies conducted in Europe and the United States have highlighted that patients using denosumab showed better adherence and persistence to treatment than those using oral bisphosphonates [20–23].

Apart from two studies in Taiwan, there is a lack of research assessing how treatment persistence and compliance could differ between patients prescribed either denosumab or oral bisphosphonates in the Asia–Pacific region [24, 25]. Medication persistence and compliance may vary between countries due to differences in the healthcare system, drug reimbursement, local treatment guidelines and patient preferences [26], which in turn could impact the effectiveness of osteoporosis treatment. In order to better inform treatment decisions and improve outcomes among women with osteoporosis in the Asia–Pacific region [27], we conducted a prospective, multicenter study with a long follow-up period (up to 24 months) between 2019 and 2023. In this interim analysis, we aimed to compare real-world medication persistence and compliance between postmenopausal women prescribed denosumab or alendronate for osteoporosis treatment over 12 months. In addition, adverse events (AEs) and changes in BMD were assessed between the two treatment groups.

## Materials and methods

### Study design and study population

This was a prospective cohort study conducted across 33 sites in 5 Asia–Pacific territories. This interim analysis included data from 25 June 2019 to 28 July 2021. The numbers of sites were 11, 6, 5, 7, and 4 in Australia, Taiwan, South Korea, Hong Kong, and Singapore, respectively. Patients were assigned to either denosumab, or alendronate group based on the medication they were prescribed for osteoporosis treatment. Data was collected at enrollment and subsequently at the 12-month visit. The schematic diagram of the study design is presented in Supplementary Fig. 1, while participating territories and sites are presented in Supplementary Fig. 2.

To be eligible for study inclusion, postmenopausal women must have received at least one prescription for denosumab or weekly oral alendronate prior to informed consent and be enrolled within four weeks after receiving the first denosumab injection or first prescription of weekly alendronate for the current course of treatment. The exclusion criteria included contraindications for treatment with both denosumab and alendronate based on approved local prescribing information (e.g., hypocalcemia, hypersensitivity), enrollment in another study involving any investigational procedure, device or drug within the preceding 6 months, as well as any kind of disorder which may compromise the ability of the patient to provide written informed consent.

Informed consent was obtained from all individual participants included in the study The study protocol received ethical approval from the Institutional Review Board of all local sites.

### Data collection

Baseline information including demographics, body mass index (BMI), smoking status, history of falls, history of fractures, current and prior treatment for osteoporosis, BMD measurements, bone densitometer model, reimbursement status, and AEs were collected at enrollment. BMD measurements and AEs were subsequently collected at routine 12-month visits. Spontaneous AEs reported in both denosumab and alendronate groups were coded in accordance with the Medical Dictionary for Regulatory Activities (MedDRA), version 22 [28].

### Calculation of treatment persistence and compliance

Patients were deemed to be persistent with denosumab if they received the second injection within 8 months or 240 days (180 days as scheduled and 60 days of allowed gap) after the first injection. The formula used to calculate the number of days between the first and second denosumab injection (gap period) was: ‘Gap period for denosumab = Date of administration (second injection of denosumab)–Date of administration (first injection of denosumab)’.

Patients were deemed to be persistent with alendronate if there was no gap period of more than 60 days during the 12-month period. Each gap period between alendronate dispensation dates was calculated 12 months (or 365 days) from the date of first dispensation. The formula used to calculate the gap period based on alendronate dispensation dates and quantity supplied was: ‘Gap period for alendronate = Date of dispensation–Date of previous dispensation – 7 days × Quantity of dispensation (number of 70 mg tablets).’

In other published studies examining persistence with osteoporosis therapies, a permissible treatment gap period of 60 days (or 8 weeks) was similarly used for denosumab and alendronate [20, 22, 23, 29]. In addition, switching treatment was considered as discontinuation of initially prescribed therapy in our study.

Participants were considered to be compliant with denosumab treatment if they received two injections within a 12-month period (i.e., second injection within 365 days from the first injection). Compliance at 12-month for weekly alendronate was measured using the medication possession ratio (MPR), which was calculated using the formula: ‘MPR = Total number of days on therapy (7 days multiplied by the quantity of dispensation) / Length of follow-up (365 days)’. Patients with an MPR ≥ 0.80 were deemed to be compliant with treatment. The definition of compliance in our study was consistent with other similar studies examining compliance with osteoporosis therapies [20, 29].

### Statistical analysis

Baseline patient characteristics were summarized using descriptive statistics. A sample size of 77 participants per treatment group was targeted for each territory, which allowed the study to have 80% power (with type 1 error rate of 0.05) to detect a significant difference in 12-month persistence between the treatment groups in each territory, assuming a dropout rate of 10%. Based on an observational real-world study, the 12-month persistence of denosumab and alendronate were estimated to be 70% and 45%, respectively [27]. Continuous variables were summarized using mean, standard deviation (SD), median and interquartile range (IQR), while categorical variables were presented using frequency and proportion. When comparing between groups, either chi-square test or Fisher’s exact test was used for categorical variables. For continuous variables, either Student’s t-test or Mann–Whitney U test was used.

The 12-month persistence and compliance were calculated for all patients and compared between treatment groups. Multivariable logistic regression was also used to examine the association between 12-month persistence (or compliance) and treatment groups. Based on clinical judgment and baseline characteristics found to be significantly different between the treatment groups, independent variables including age, history of fracture, measurement of BMD, prior treatment for osteoporosis, and prior treatment with glucocorticoids were added into the model for adjustment. If collinearity was encountered, key variables (i.e., age and history of fracture) were first retained in the model, while other variables were then added based on statistical differences observed between treatment groups (i.e., variables with lower *P*-values will be prioritized for adjustment in the model). If necessary, continuous variables were also categorized. Sensitivity analysis for the 12-month persistence across territories was carried out by further adjusting for baseline drug reimbursement status when there were at least 10% subjects in reimbursement categories for both treatment groups. Findings were presented using adjusted odds ratio (aOR) and 95% confidence interval (CI).

The change in BMD T-scores was measured both as an absolute change and as a percentage change from baseline. In addition, multivariable linear regression was conducted to examine the association between change in BMD T-scores and treatment groups. Independent variables included age, history of fracture, baseline BMD (at respective sites), prior treatment for osteoporosis, prior treatment with glucocorticoids, model of measuring device, history of falls, BMI, smoking status, and ethnicity. For each site (lumbar spine, total hip, femoral neck), findings were reported using regression coefficient and 95% CI.

All statistical tests were two-sided, and a *P*-value < 0.05 was considered statistically significant. Data analyses were performed using SAS software, version 9.4.

## Results

### Enrollment and baseline characteristics

The study enrolled a total of 686 eligible patients from 33 sites across five territories, with 156 in Australia, 152 in Taiwan, 154 in South Korea, 155 in Hong Kong, and 69 in Singapore. Among the enrolled participants, 519 were from hospitals, 148 from clinics, and 19 from research centers. 580 patients completed their 12-month visit and were included in the analysis (301 in denosumab group and 279 in alendronate group) (Supplementary Table 1).

The mean age of all patients was 69.8 ± 9.0 years, ranging from 45 to 96 years old (Table 1). On average, patients who received denosumab (70.7 ± 9.3 years) were significantly older, compared with those prescribed alendronate (68.9 ± 8.5 years; *P* = 0.013). Moreover, there was a significantly greater proportion of older patients (≥ 75 years) in the denosumab group (32.2%), compared with alendronate group (24.0%; *P* = 0.028). There were also significant differences in prior treatment with oral glucocorticoids and prior treatment for osteoporosis between the treatment groups. A higher proportion of patients in the denosumab group (17.9%) received previous treatment with oral glucocorticoids, compared with alendronate group (6.1%; *P* < 0.001). Similarly, there was a greater proportion of participants in the denosumab group who received prior osteoporosis treatment (55.8% vs. 34.8%; *P* < 0.001). A greater percentage of patients in the denosumab group had received parenteral bisphosphonate therapy (25.6% vs. 14.4%; *P* = 0.033). On the contrary, a greater proportion of those in the alendronate group received estrogen and menopausal hormone therapy (16.5% vs. 6.5%; *P* = 0.010). Furthermore, a larger proportion of patients prescribed denosumab (30.9%) were without baseline BMD measurements, compared with those prescribed with alendronate (18.6%; *P* < 0.001). There were no significant differences between the treatment groups for other baseline demographics and patient characteristics. Both treatment groups were similar in ethnicity, BMI, smoking status, history of falls within the last year, history of fracture, parents’ history of fracture, baseline BMD measurements, and drug reimbursement status.

**Table 1:** Demographics and clinical characteristics stratified by treatment group

Characteristic	Total (N = 580)		Denosumab (n = 301)		Alendronate (n = 279)		*P*-value^a^
	N	%	n	%	n	%	
Age, n (%)^a^							
Mean (SD)	69.8 (9.0)		70.7 (9.3)		68.9 (8.5)		0.013*^b^
Min; Max	45.0; 96.0		45.0; 96.0		46.0; 93.0		
Median (IQR)	69.0 (64.0; 76.0)		70.0 (65.0; 77.0)		69.0 (63.0; 74.0)		
Age group, n (%)							
≥ 75 years old	164	28.3%	97	32.2%	67	24.0%	0.028*
< 75 years old	416	71.7%	204	67.8%	212	76.0%	
Ethnicity, n (%)^b^							
Caucasian	108	18.6%	49	16.3%	59	21.1%	0.279^c^
Chinese	325	56.0%	173	57.5%	152	54.5%	
Korean	124	21.4%	65	21.6%	59	21.1%	
Malay	2	0.3%	2	0.7%	0	0.0%	
Indian	3	0.5%	3	1.0%	0	0.0%	
Others	18	3.1%	9	3.0%	9	3.2%	
Body mass index (kg/m^2^)							
Mean (SD)	23.8 (4.6)		23.7 (4.6)		23.9 (4.5)		0.522
Min; Max	14.6; 53.0		14.6; 44.6		15.8; 53.0		
Median (IQR)	23.2 (20.6; 26.0)		23.1 (20.4; 26.0)		23.2 (21.0; 26.0)		
Missing, n (%)	3 (0.5%)		1 (0.3%)		2 (0.7%)		
Smoking status, n (%)							
Current smoker	9	1.6%	3	1.0%	6	2.2%	0.446^c^
Former smoker	42	7.2%	20	6.6%	22	7.9%	
Never smoker	529	91.2%	278	92.4%	251	90.0%	
Falls within last year, n (%)							
Yes	155	26.7%	73	24.3%	82	29.4%	0.162
One	116	74.8%	58	79.5%	58	70.7%	
Two to five	36	23.2%	14	19.2%	22	26.8%	
Five or more	3	1.9%	1	1.4%	2	2.4%	
No	425	73.3%	228	75.7%	197	70.6%	
Prior treatment with oral glucocorticoids, n (%)							
Yes	71	12.2%	54	17.9%	17	6.1%	< .001*
No	509	87.8%	247	82.1%	262	93.9%	
Prior treatment for osteoporosis, n (%)							
Yes^d^	265	45.7%	168	55.8%	97	34.8%	< .001*
Oral bisphosphonate therapy	155	58.5%	102	60.7%	53	54.6%	0.334
Calcitriol	13	4.9%	8	4.8%	5	5.2%	1.000^c^
Denosumab	62	23.4%	42	25.0%	20	20.6%	0.417
Injectable bisphosphonate therapy	57	21.5%	43	25.6%	14	14.4%	0.033*
Selective estrogen receptor modulators	33	12.5%	16	9.5%	17	17.5%	0.057
Calcitonin or elcatonin	5	1.9%	4	2.4%	1	1.0%	0.655^c^
Parathyroid hormone or teriparatide	24	9.1%	17	10.1%	7	7.2%	0.428
Strontium	13	4.9%	10	6.0%	3	3.1%	0.385^c^
Estrogen or hormone therapy	27	10.2%	11	6.5%	16	16.5%	0.010*
Other types^e^	18	6.8%	10	6.0%	8	8.2%	0.474
No	315	54.3%	133	44.2%	182	65.2%	
History of fracture, n (%)							
Yes	267	46.0%	135	44.9%	132	47.3%	0.552
Site of fracture^f^							
Hip	35	13.1%	20	14.8%	15	11.4%	0.403
Vertebral	129	48.3%	70	51.9%	59	44.7%	0.242
Wrist	52	19.5%	23	17.0%	29	22.0%	0.309
Other sites	95	35.6%	49	36.3%	46	34.8%	0.805
Type of fracture^g^							
Osteoporotic	248	92.9%	123	91.1%	125	94.7%	0.255
Other types	32	12.0%	19	14.1%	13	9.8%	0.288
No	313	54.0%	166	55.1%	147	52.7%	
Parent history of fracture, n (%)							
Yes	73	12.6%	41	13.6%	32	11.5%	0.435
No	507	87.4%	260	86.4%	247	88.5%	
Measurement of bone mineral density (BMD)							
Yes, n (%)	434	74.8%	207	68.8%	227	81.4%	< .001*
Lumbar spine^h^							
T-score							
Mean (SD)	− 2.3 (1.2)		− 2.3 (1.3)		− 2.3 (1.2)		0.638
Min; Max	− 5.5; 3.5		− 5.4; 2.6		− 5.5; 3.5		
Median (IQR)	− 2.5 (− 3.1; − 1.7)		− 2.5 (− 3.1; − 1.7)		− 2.5 (− 3.0; − 1.7)		
Total hip^i^							
T-score							
Mean (SD)	− 2.0 (0.8)		− 2.1 (0.8)		− 2.0 (0.9)		0.232
Min; Max	− 5.5; 1.2		− 4.3; 0.0		− 5.5; 1.2		
Median (IQR)	− 2.0 (-2.5; -1.5)		− 2.1 (− 2.5; − 1.6)		− 2.0 (− 2.5; − 1.4)		
Femoral neck^j^							
T-score							
Mean (SD)	− 2.4 (0.8)		− 2.4 (0.8)		− 2.3 (0.8)		0.328
Min; Max	− 5.3; 2.4		− 4.5; -0.1		− 5.3; 2.4		
Median (IQR)	− 2.4 (-2.8; -1.9)		− 2.5 (− 2.9; − 2.0)		− 2.4 (− 2.8; − 1.9)		
No, n (%)	145	25.0%	93	30.9%	52	18.6%	
Missing, n (%)	1	0.2%	1	0.3%	0	0.0%	
Reimbursement status, n (%)							
Fully reimbursed	213	36.7%	120	39.9%	93	33.3%	0.194
Partially reimbursed	206	35.5%	98	32.6%	108	38.7%	
Self-funded	159	27.4%	81	26.9%	78	28.0%	
Missing	2	0.3%	2	0.7%	0	0.0%	

^a^Unless otherwise specified, p-values were generated by Mann–Whitney U test for continuous variables and chi-square test for categorical variables

^b^Student’s t-test was used to compare the mean age of patients on denosumab and those on alendronate

^c^Fisher’s exact test was used to compare patients on denosumab and those on alendronate

^d^A patient may have received multiple therapies. Hence, the sum of percentages for subcategories may exceed 100%

^e^Include records with unknown name of therapy

^f^A patient may have multiple sites of fracture. Hence, the sum of percentages for subcategories may exceed 100%

^g^A patient may have multiple types of fracture. Hence, the sum of percentages for subcategories may exceed 100%

^h^The number of patients with measurement of bone mineral density at lumbar spine is 403, among which 194 patients were on denosumab, and 209 patients were on alendronate

^i^The number of patients with measurement of bone mineral density at total hip is 398, among which 188 patients were on denosumab, and 210 patients were on alendronate

^j^The number of patients with measurement of bone mineral density at femoral neck is 425, among which 201 patients were on denosumab, and 224 patients were on alendronate

^*^p < 0.05

*BMD* bone mineral density, *IQR* interquartile range, *Max* maximum, *Min* minimum, *SD* standard deviation

### Overall 12-month medication persistence and compliance

The overall osteoporosis treatment persistence and compliance were 72.4% and 70.7%, respectively. Patients on denosumab demonstrated significantly higher levels of persistence (82.7%) and compliance (86.0%), in contrast to those on alendronate (61.3% and 54.1%, respectively; P < 0.001) (Table 2).

**Table 2:** Osteoporosis treatment persistence and compliance at 12 months

	Total (N = 580)	Denosumab (n = 301)	Alendronate (n = 279)	*P*-value^c^
Persistence, n (%)^a^	420 (72.4%)	249 (82.7%)	171 (61.3%)	< .001*
Compliance, n (%)^b^	410 (70.7%)	259 (86.0%)	151 (54.1%)	< .001*

^a^For denosumab, persistence at 12 months is defined as the patient receiving her second injection of denosumab within 8 months (6 months as scheduled and 2 months of allowed gap) after the first injection. For alendronate, persistence at 12 months is defined as remaining on therapy with no gap period > 60 days during the 12-month period (gap period calculated based on alendronate dispensation dates and quantity supplied)

^b^For denosumab, compliance at 12 months is defined as having received 2 injections within 12 months. For alendronate, compliance at 12 months is defined as the medication possession ratio (total days on therapy divided by 365 days) ≥ 0.80

^c^*P*-values were generated by chi-square test for categorical variables

^*^P < 0.05

*IQR* interquartile range, *Max* maximum, *Min* minimum, *SD* standard deviation, - not applicable

Across all territories, treatment group and lower age were significantly associated with treatment persistence at 12 months (Table 3), and this association persisted after adjusting for baseline reimbursement status in the sensitivity analysis (Supplementary Table 2). Patients in the denosumab group were about three-fold more likely to be persistent, compared with those in the alendronate group (aOR = 3.08; 95% CI 2.04–4.63) (Table 3). Older patients had lower odds of treatment persistence (aOR = 0.97; 95% CI 0.95–1.00, *P* = 0.018). History of fracture, baseline measurement of BMD, prior treatment for osteoporosis, and prior oral glucocorticoid treatment were not significantly associated with treatment persistence (*P* > 0.05). When regression was performed for each territory, being in the denosumab group was significantly associated with treatment persistence at 12 months for Australia (aOR = 19.42; 95% CI 7.13–52.89) and Singapore (aOR = 7.83; 95% CI 1.58–38.84) only.

**Table 3:** Adjusted odds ratios and 95% confidence intervals for osteoporosis treatment persistence at 12 months using multivariable logistic regression

Territory	Adjusted odds ratio^a^	95% CI	*P*-value	Reference group^b^
All territories, (N = 580)				
Denosumab group	3.08	(2.04–4.63)	< .001*	alendronate
Age	0.97	(0.95–1.00)	0.018*	-
History of fracture	0.81	(0.55–1.19)	0.281	no
Measurement of BMD	0.96	(0.61–1.51)	0.867	no
Prior treatment for osteoporosis	1.44	(0.97–2.14)	0.068	no
Prior treatment with oral glucocorticoids	0.69	(0.37–1.27)	0.229	no
Australia, (n = 142)				
Denosumab group	19.42	(7.13–52.89)	< .001*	alendronate
Age	0.94	(0.89–0.99)	0.022*	-
History of fracture	1.19	(0.51–2.77)	0.686	no
Measurement of BMD	1.39	(0.38–5.12)	0.622	no
Prior treatment for osteoporosis	0.50	(0.19–1.30)	0.155	no
Prior treatment with oral glucocorticoids	1.25	(0.38–4.09)	0.716	no
Taiwan, (n = 109)				
Denosumab group	1.62	(0.56–4.75)	0.376	alendronate
Age	1.01	(0.95–1.07)	0.819	-
History of fracture	0.46	(0.12–1.72)	0.246	no
Measurement of BMD	3.40	(0.80–14.45)	0.097	no
Prior treatment for osteoporosis	2.95	(0.63–13.69)	0.168	no
Prior treatment with oral glucocorticoids	5.85	(0.32–106.62)	0.233	no
South Korea, (n = 124)				
Denosumab group	1.81	(0.60–5.45)	0.291	alendronate
Age	0.94	(0.88–1.00)	0.048*	-
History of fracture	0.48	(0.16–1.45)	0.195	no
Measurement of BMD	2.21	(0.63–7.77)	0.218	no
Prior treatment for osteoporosis	2.78	(0.96–8.10)	0.061	no
Prior treatment with oral glucocorticoids	0.46	(0.13–1.64)	0.229	no
Hong Kong, (n = 141)				
Denosumab group	1.45	(0.59–3.57)	0.418	alendronate
Age	1.00	(0.95–1.04)	0.858	-
History of fracture	1.05	(0.45–2.48)	0.904	no
Measurement of BMD	1.68	(0.69–4.12)	0.257	no
Prior treatment for osteoporosis	2.18	(0.82–5.83)	0.121	no
Prior treatment with oral glucocorticoids	0.17	(0.03–0.90)	0.037*	no
Singapore, (n = 64)				
Denosumab group	7.83	(1.58–38.84)	0.012*	alendronate
Age	0.93	(0.84–1.04)	0.183	-
History of fracture	0.49	(0.12–2.00)	0.318	no
Measurement of BMD	1.44	(0.29–7.06)	0.653	no
Prior treatment for osteoporosis	0.96	(0.23–3.98)	0.949	no
Prior treatment with oral glucocorticoids	5.05	(0.14–183.87)	0.378	no

^a^Adjusted odds ratios were generated using multivariable logistic regression models. Treatment persistence was used as the dependent variable, while adjusting for treatment group, age, history of fracture, measurement of BMD, prior treatment for osteoporosis, and prior treatment with oral glucocorticoids

^b^Treatment group is categorized as patients on denosumab and those on alendronate. History of fracture includes with fracture and without fracture. Measurement of BMD includes with BMD measurement and without. Prior treatment for osteoporosis includes using and not using osteoporosis drug before starting denosumab or alendronate treatment. Prior treatment with oral glucocorticoids includes using and not using oral glucocorticoids before starting denosumab or alendronate treatment

^*^P < 0.05

*BMD* bone mineral density, *CI* confidence interval, - not applicable

Across all territories, treatment group, history of fracture, prior treatment for osteoporosis and prior oral glucocorticoid treatment were significantly associated with treatment compliance at 12 months (Table 4). Patients in the denosumab group were about five-fold more likely to be compliant, compared with those in the alendronate group (aOR = 5.32; 95% CI 3.45–8.21). Patients who received prior treatment for osteoporosis before study enrollment were 1.63 times (95% CI 1.09–2.45) more likely to be compliant with treatment. However, patients with a history of fracture (aOR = 0.61; 95% CI 0.41–0.90) and who received prior oral glucocorticoid treatment (aOR = 0.48; 95% CI 0.26–0.90) were less likely to be compliant. Age and baseline measurement of BMD were not significantly associated with treatment compliance (*P* > 0.05). When regression was performed for each territory, being in the denosumab group was significantly associated with treatment compliance at 12 months for all territories, except Hong Kong (aOR = 1.82; 95% CI 0.64– 5.13).

**Table 4:** Adjusted odds ratios and 95% confidence intervals for osteoporosis treatment compliance at 12 months using multivariable logistic regression

Territory	Adjusted odds ratio ^a^	95% CI	*P*-value	Reference group^b^
All territories, (N = 580)				
Denosumab group	5.32	(3.45–8.21)	< .001*	alendronate
Age	0.99	(0.96–1.01)	0.213	-
History of fracture	0.61	(0.41–0.90)	0.013*	no
Measurement of BMD	0.81	(0.51–1.31)	0.394	no
Prior treatment for osteoporosis	1.63	(1.09–2.45)	0.018*	no
Prior treatment with oral glucocorticoids	0.48	(0.26–0.90)	0.022*	no
Australia, (n = 142)				
Denosumab group	34.85	(11.73–103.52)	< .001*	alendronate
Age	0.96	(0.91–1.02)	0.196	-
History of fracture	0.86	(0.34–2.17)	0.746	no
Measurement of BMD	0.94	(0.23–3.83)	0.933	no
Prior treatment for osteoporosis	0.51	(0.18–1.44)	0.203	no
Prior treatment with oral glucocorticoids	0.97	(0.27–3.42)	0.956	no
Taiwan, (n = 109)				
Denosumab group	5.32	(1.96–14.48)	0.001*	alendronate
Age	1.01	(0.96–1.07)	0.646	-
History of fracture	0.40	(0.13–1.27)	0.119	no
Measurement of BMD	1.52	(0.43–5.38)	0.514	no
Prior treatment for osteoporosis	2.42	(0.66–8.92)	0.184	no
Prior treatment with oral glucocorticoids	1.93	(0.29–13.07)	0.500	no
South Korea, (n = 124)				
Denosumab group	3.45	(1.08–10.97)	0.036*	alendronate
Age	0.96	(0.90–1.02)	0.150	-
History of fracture	0.47	(0.15–1.50)	0.204	no
Measurement of BMD	2.26	(0.63–8.04)	0.210	no
Prior treatment for osteoporosis	3.76	(1.25–11.25)	0.018*	no
Prior treatment with oral glucocorticoids	0.51	(0.13–1.94)	0.320	no
Hong Kong, (n = 141)				
Denosumab group	1.82	(0.64–5.13)	0.260	alendronate
Age	1.00	(0.95–1.05)	0.989	-
History of fracture	1.20	(0.45–3.19)	0.710	no
Measurement of BMD	1.70	(0.62–4.66)	0.305	no
Prior treatment for osteoporosis	1.46	(0.50–4.29)	0.491	no
Prior treatment with oral glucocorticoids	0.13	(0.02–0.76)	0.023*	no
Singapore, (n = 64)				
Denosumab group	12.30	(2.25–67.35)	0.004*	alendronate
Age	0.96	(0.86–1.07)	0.442	-
History of fracture	0.67	(0.15–2.94)	0.597	no
Measurement of BMD	1.25	(0.24–6.61)	0.791	no
Prior treatment for osteoporosis	0.80	(0.18–3.53)	0.768	no
Prior treatment with oral glucocorticoids	0.27	(0.02–4.04)	0.339	no

^a^Adjusted odds ratios were generated using multivariable logistic regression models. Treatment persistence was used as the dependent variable, while adjusting for treatment group, age, history of fracture, measurement of BMD, prior treatment for osteoporosis, and prior treatment with oral glucocorticoids

^b^Treatment group is categorized as patients on denosumab and those on alendronate. History of fracture includes with fracture and without fracture. Measurement of BMD includes with BMD measurement and without. Prior treatment for osteoporosis includes using and not using osteoporosis drug before starting denosumab or alendronate treatment. Prior treatment with oral glucocorticoids includes using and not using oral glucocorticoids before starting denosumab or alendronate treatment

^*^P < 0.05

*BMD* bone mineral density, *CI* confidence interval, - not applicable

### Changes in BMD T-score from baseline at 12-month visit

In the denosumab group, the mean baseline BMD T-score for lumbar spine, total hip, and femoral neck were − 2.4, − 2.0, and − 2.4, respectively (Supplementary Table 3). After 12 months, T-scores for the respective sites improved to − 2.1, − 1.9 and − 2.3. Change in T-score from baseline was the greatest for the lumbar spine (10.5%) and least for the femoral neck (2.6%). In the alendronate group, the mean baseline T-score for lumbar spine, total hip and femoral neck were similar (− 2.4, − 1.9, and − 2.3, respectively), when compared with denosumab group. After 12 months, T-scores for the respective sites improved to − 2.2, − 1.8, and − 2.2. Change in T-score from baseline was the greatest for the lumbar spine (13.0%). Treatment type was not significantly associated with mean changes and percentage changes in T-scores from baseline for all sites (Supplementary Tables 4 and 5). However, baseline BMD was consistently negatively associated with changes in T-scores. A lower baseline BMD tends to result in greater percentage improvement in T-score.

### Adverse events at 12-month visit

In both treatment groups, similar proportions of patients experienced adverse events (23.9% in denosumab group vs. 21.5% in alendronate group) (Table 5). There was no significant difference in the AE outcome between the two treatment groups (P = 0.205). The AEs reported were considered to be unrelated to the treatment in a significantly higher proportion of patients in the denosumab group, compared with alendronate group (86.5% vs. 54.6%, P < 0.001). Meanwhile, a significantly greater proportion of patients in the alendronate group, in contrast to those in the denosumab group, experienced treatment interruption (4.0% vs. 17.6%) and withdrawal (2.4% vs. 11.1%; P < 0.001). Gastrointestinal disorders were more frequently reported in the alendronate group than in the denosumab group (30.6% vs. 8.7%, P < 0.001). On the other hand, AEs (n = 27) falling within the classification of ‘injury, poisoning, and procedural complications’ were more commonly reported in the denosumab group (16.7% vs. 5.6%, P = 0.008). Within this category, 16 AEs were bone and joint injuries, 9 AEs were injuries not elsewhere classified (non-specific), while 2 AEs were considered procedural-related injuries and complications. None of these 27 events were related to denosumab or alendronate treatment. The occurrence of other AEs was also similar between the two treatment groups. Hospitalization was more common in the denosumab group than in the alendronate group (29.4% vs. 15.7%, P = 0.014). There was no significant difference in life-threatening events or death between both treatment groups.

**Table 5:** Adverse events at 12 months by treatment group

Type	Total (N = 234)		Denosumab (n = 126)		Alendronate (n = 108)		*P*-value^b^
	N	%	n	%	n	%	
Patient count, n (%)^a^	580	100%	301	100%	279	100%	0.488
No adverse event	448	77.2%	229	76.1%	219	78.5%	
With adverse event	132	22.8%	72	23.9%	60	21.5%	
One	83	62.9%	49	68.1%	34	56.7%	
Two	20	15.2%	8	11.1%	12	20.0%	
Three or more	29	22.0%	15	20.8%	14	23.3%	
Relationship to treatment, n (%)							
Not related	168	71.8%	109	86.5%	59	54.6%	< .001*
Related	27	11.5%	6	4.8%	21	19.4%	
Possibly related	23	9.8%	5	4.0%	18	16.7%	
Unlikely related	16	6.8%	6	4.8%	10	9.3%	
Action taken with study treatment, n (%)							
No action taken	186	79.5%	112	88.9%	74	68.5%	< .001*^c^
Treatment withdrawn	24	10.3%	5	4.0%	19	17.6%	
Treatment interrupted	15	6.4%	3	2.4%	12	11.1%	
Not applicable	7	3.0%	4	3.2%	3	2.8%	
Change medication	1	0.4%	1	0.8%	0	0.0%	
Delayed	1	0.4%	1	0.8%	0	0.0%	
Outcome, n (%)							
Recovered / Resolved	176	75.2%	98	77.8%	78	72.2%	0.205^c^
Not recovered / Not resolved	44	18.8%	18	14.3%	26	24.1%	
Fatal	8	3.4%	5	4.0%	3	2.8%	
Unknown	5	2.1%	4	3.2%	1	0.9%	
Recovered / Resolved with sequelae	1	0.4%	1	0.8%	0	0.0%	
Adverse events, n (%)							
Gastrointestinal disorders	44	18.8%	11	8.7%	33	30.6%	< .001*
Infections and infestations	32	13.7%	20	15.9%	12	11.1%	0.291
Musculoskeletal and connective tissue disorders	28	12.0%	15	11.9%	13	12.0%	0.975
Injury, poisoning, and procedural complications^d^	27	11.5%	21	16.7%	6	5.6%	0.008*
General disorders and administration site conditions	20	8.5%	9	7.1%	11	10.2%	0.407
Nervous system disorders	17	7.3%	10	7.9%	7	6.5%	0.669
Metabolism and nutrition disorders	10	4.3%	7	5.6%	3	2.8%	0.348^c^
Respiratory, thoracic, and mediastinal disorders	10	4.3%	4	3.2%	6	5.6%	0.520^c^
Skin and subcutaneous tissue disorders	9	3.8%	4	3.2%	5	4.6%	0.736^c^
Neoplasms benign, malignant, and unspecified (including cysts and polyps)	6	2.6%	3	2.4%	3	2.8%	1.000^c^
Cardiovascular disorders	10	4.3%	6	4.8%	4	3.7%	0.756^c^
Ear and labyrinth disorders	4	1.7%	4	3.2%	0	0.0%	0.126^c^
Investigations	4	1.7%	3	2.4%	1	0.9%	0.626^c^
Psychiatric disorders	3	1.3%	2	1.6%	1	0.9%	1.000^c^
Eye disorders	2	0.9%	2	1.6%	0	0.0%	0.501^c^
Hepatobiliary disorders	2	0.9%	1	0.8%	1	0.9%	1.000^c^
Renal and urinary disorders	2	0.9%	1	0.8%	1	0.9%	1.000^c^
Blood and lymphatic system disorders	1	0.4%	0	0.0%	1	0.9%	0.462^c^
Surgical and medical procedures	1	0.4%	1	0.8%	0	0.0%	1.000^c^
Missing	2	0.9%	2	1.6%	0	0.0%	0.501^c^
Serious adverse events, n (%)							
Hospitalization	54	23.1%	37	29.4%	17	15.7%	0.014*
Gastrointestinal disorders	4	7.4%	1	2.7%	3	17.6%	0.087^c^
Infections and infestations	11	20.4%	7	18.9%	4	23.5%	0.726^c^
Musculoskeletal and connective tissue disorders	3	5.6%	3	8.1%	0	0.0%	0.544^c^
Injury, poisoning, and procedural complications	13	24.1%	11	29.7%	2	11.8%	0.189^c^
General disorders and administration site conditions	3	5.6%	1	2.7%	2	11.8%	0.230^c^
Nervous system disorders	3	5.6%	2	5.4%	1	5.9%	1.000^c^
Metabolism and nutrition disorders	3	5.6%	3	8.1%	0	0.0%	0.544^c^
Respiratory, thoracic, and mediastinal disorders	1	1.9%	1	2.7%	0	0.0%	1.000^c^
Neoplasms benign, malignant, and unspecified (including cysts and polyps)	4	7.4%	2	5.4%	2	11.8%	0.582^c^
Cardiovascular disorders	5	9.3%	3	8.1%	2	11.8%	0.645^c^
Ear and labyrinth disorders	1	1.9%	1	2.7%	0	0.0%	1.000^c^
Investigations	1	1.9%	0	0.0%	1	5.9%	0.315^c^
Hepatobiliary disorders	1	1.9%	1	2.7%	0	0.0%	1.000^c^
Surgical and medical procedures	1	1.9%	1	2.7%	0	0.0%	1.000^c^
Death	5	2.1%	4	3.2%	1	0.9%	0.377^c^
Other medically important event^e^	2	0.9%	2	1.6%	0	0.0%	0.501^c^
Life-threatening	2	0.9%	2	1.6%	0	0.0%	0.501^c^
Significant disability	1	0.4%	1	0.8%	0	0.0%	1.000^c^
Congenital anomaly or birth defect	0	0.0%	0	0.0%	0	0.0%	-

^a^Adverse events were grouped based on their System Organ Classes derived from the Medical Dictionary for Regulatory Activities Version 22

^b^Unless otherwise specified, *P-*values were generated by chi-square test for categorical variables

^c^Fisher’s exact test was used to determine the association between variables related to adverse events and treatment groups

^d^In the denosumab group, 21 events included 11 cases of bone and joint injuries, 8 cases of injuries not elsewhere classified, and 2 cases of procedural related injuries and complications. In the alendronate group, 6 events included 5 cases of bone and joint injuries, and 1 case of injuries not elsewhere classified

^e^Includes neoplasms

*P < 0.05

- not applicable

## Discussion

To the best of our knowledge, this was the first multicenter prospective cohort study investigating the persistence and compliance of denosumab compared with weekly alendronate among postmenopausal women with osteoporosis in the Asia–Pacific region. At baseline, patients in the denosumab group were older and more likely to have received prior oral glucocorticoids or osteoporosis treatment than those in the alendronate group. Patients prescribed denosumab displayed significantly higher persistence and compliance with their therapy in the following 12 months, compared with those who received alendronate. The significant association between treatment type and drug utilization persisted in our study, even after adjustment for potential confounders. Although BMD T-scores improved in both study groups after 12 months, we did not detect a significant association between treatment type and change in BMD T-score at the lumbar spine, total hip, or femoral neck.

Patients in the two treatment groups were largely similar in their demographics and clinical characteristics. However, the study results showed that patients prescribed denosumab were significantly older and more likely to have received prior osteoporosis treatment, compared with those in the alendronate group. This finding was in line with a similar study conducted in Canada that examined patient characteristics and the usage of osteoporosis therapies [30], and could be attributed to physicians following osteoporosis treatment guidelines and drug reimbursement policies in the territories where the study was conducted. For instance, guidelines from the Osteoporosis Society of Hong Kong (OSHK) have recommended denosumab as an alternative therapy for postmenopausal patients with contraindications to oral bisphosphonate therapies, unable to tolerate oral or intravenous bisphosphonate therapies, or who had suboptimal clinical responses to other antiresorptive therapies [31, 32]. Thus, it is not surprising that most women may be prescribed oral bisphosphonates as first-line treatment (in accordance with guidelines), and subsequently switched to another treatment such as denosumab if they do not respond, experience intolerable side effects, or have difficulty with treatment adherence. Furthermore, studies have suggested that the effectiveness of bisphosphonates in increasing BMD may diminish over longer treatment periods [33]. This may prompt physicians to subsequently prescribe alternative therapies such as denosumab, to maintain or further improve BMD in patients with osteoporosis [24, 25, 33].

Our study results indicated that patients receiving denosumab injection treatment demonstrated higher persistence and compliance rates, compared with those receiving weekly oral alendronate treatment. This was supported by previous studies which had also reported the advantages of denosumab over alendronate in persistence and compliance [20, 23]. Denosumab has a dosing schedule of every 6 months, making it theoretically more convenient than alendronate, which requires weekly or daily administration [15]. The frequent occurrence of gastrointestinal side effects and specific requirements for the administration of oral bisphosphonates, such as taking them on an empty stomach with water (at least 30 min before food) and maintaining an upright position for at least 30 min [31, 32], have been identified as major factors contributing to discontinuation of oral bisphosphonates [34]. Another factor that may contribute to higher adherence and compliance with denosumab is the difference in pharmacological guidance between the two medications provided to patients. Prescribers recommend not discontinuing denosumab abruptly, as this may induce new clinical vertebral fractures if treatment with a bisphosphonate is not initiated afterward [35].

Earlier research conducted in Asia and before the COVID-19 pandemic had reported generally higher persistence and compliance with oral bisphosphates, compared with our study. A study in Singapore, which examined the use of alendronate and risedronate, reported 1-year persistence and compliance of 69% and 72%, respectively [36]. Another Korean study had estimated 1-year persistence and compliance with oral bisphosphates as 63.3% and 60.1%, respectively [37]. In our study, persistence (61.3%) and compliance (54.1%) with alendronate were comparatively lower. During the COVID pandemic, it is possible that treatment for less life-threatening chronic illnesses (such as osteoporosis) was postponed or delayed for various reasons, such as the implementation of strict lockdown measures or patients’ concerns about the risk of infection when visiting a healthcare facility [38, 39]. These may have negatively impacted treatment persistence and compliance.

Interestingly, patients prescribed denosumab in our study had persistence and compliance rates that were comparable with those observed in pre-pandemic studies [22, 23, 29]. For example, the 12-month persistence with denosumab in a Swiss retrospective study was 83%, which closely resembled our study’s findings (82.7%) [23]. Another multicenter prospective European study revealed 12-month compliance rates ranging from 82.7 to 89.3% across four countries [22], also like our study's compliance rate of 86.0%. Since denosumab treatment requires fewer healthcare facility visits due to its longer dosing interval (compared with alendronate), its use may be less affected by the pandemic. Given the relative advantages of denosumab in treatment persistence and compliance, healthcare providers may consider its use over oral bisphosphates, especially during pandemics when healthcare utilization can be severely disrupted, and routine collection of medications becomes challenging for patients.

Although patients in the denosumab group had significantly higher treatment persistence and compliance, no significant association was detected between BMD and treatment type for all sites (i.e., lumbar spine, total hip, and femoral neck). This is likely due to the relatively small sample sizes in our study. This study was primarily designed to compare medication persistence between the two treatments as the main objective and therefore, may not have sufficient statistical power to detect a difference in BMD T-scores. In addition, the BMD could be measured using different devices, which may have led to some inconsistencies in measurements among patients. A previous meta-analysis of randomized controlled trials (RCTs) reported a significantly greater increase in BMD after 12 months with denosumab (compared with alendronate) at various skeletal sites assessed (i.e., total hip, lumbar spine, femoral neck, and trochanter) [17]. However, RCTs often have stringent study eligibility criteria which reduces the generalizability of the results. Certain patient groups such as postmenopausal women with metabolic bone diseases (e.g., hyper- or hypothyroidism and hyper- or hypoparathyroidism), or those with inflammatory conditions (e.g., rheumatoid arthritis, systemic lupus erythematosus) which necessitate treatment using glucocorticoids, may be excluded from RCTs as their concomitant medical conditions or medications could potentially interfere with the efficacy of osteoporosis drugs. It is known that long-term use of glucocorticoids reduces BMD by impairing bone formation and increasing bone resorption [40, 41]. In contrast, our study had relatively less strict inclusion and exclusion criteria, which allowed the inclusion of a wider population of patients to examine the real-world effectiveness of drug treatment in routine medical practice. A significantly higher proportion of patients in the denosumab group had a history of prior oral glucocorticoid use, compared with the alendronate group. Although this medication history was included for adjustment in the regression analyses, it was uncertain whether patients may have received glucocorticoids after study enrollment. Missing data could be another reason why our study did not detect a significant association between BMD and treatment type after 12 months. Almost a third of patients in the denosumab group and about 19% of patients in the alendronate did not have baseline BMD measurements. It is unclear why these data were missing. Some patients could have switched hospitals resulting in incomplete medical records, osteoporosis may have been diagnosed based on clinical history (e.g., history of fragility fractures), or it may have been due to logistic challenges in scheduling bone density scans during the COVID-19 pandemic. Our study findings suggested that most patients did not report any AEs over 12 months of follow-up. However, among those who did, the most common AE was gastrointestinal disorders. In particular, nearly one-third of patients in the alendronate group reported gastrointestinal issues. While alendronate treatment was not found to be associated with an increased incidence of upper gastrointestinal tract events among postmenopausal women in RCTs [42], post-marketing studies on oral bisphosphonates have highlighted gastrointestinal disorders as a key concern in patients [43]. In the real-world, esophageal and gastric side effects are commonly cited reasons for discontinuation of oral bisphosphonates [44]. This may explain the higher frequency of treatment interruption and withdrawal observed among participants receiving alendronate in our study.

In addition, our study showed that AEs concerned with bone and joint injuries and procedural-related complications were more commonly reported in patients prescribed denosumab, compared with those prescribed alendronate. However, none of these events were related to treatment. The higher proportion of patients in the denosumab group who experienced bone and joint injuries (including fractures and dislocations classified in MedDRA), and procedural-related complications may be attributed to the significantly older mean age of this group, compared with alendronate group. These injuries might necessitate surgical interventions which could help explain the higher proportion of hospitalization among patients in denosumab group. Other comorbidities present in individuals with osteoporosis can also influence the reported incidence of AEs. For example, a cross-sectional study found that a significant proportion of adults with osteoporosis had comorbid conditions such as coronary heart disease or arthritis [45]. These additional health conditions may contribute to reporting of AEs unrelated to osteoporosis treatment. As data on comorbidities were not collected in this study, it is unknown whether they were evenly distributed between the treatment groups.

Our findings should be interpreted with caution due to several limitations. Firstly, the COVID-19 pandemic and subsequent implementation of lockdowns and social distancing measures in the Asia–Pacific region considerably impacted the study enrollment process and disrupted patients’ routine medical appointments. Enrollment of patients was most affected in Singapore, with the smallest number enrolled compared with other territories. Even though missing data should be minimized using a prospective study design, there were still many patients in our study (25% in total) without baseline BMD measurement. In addition, our study findings may be influenced by differences in BMD measuring equipment and protocols across the study centers. Secondly, our study had relatively small sample sizes which may have affected our study findings. To minimize the impact from this, pooled data from all territories were analyzed. Thirdly, we observed higher than expected loss to follow-up in several territories. The proportion of patients who withdrew from our study exceeded 10% in Taiwan (28.3%) and South Korea (19.5%). Consequently, the study faced challenges in maintaining the required participant numbers (at least 70 in each treatment group) for analysis and this also limited the feasibility of conducting further subgroup analyses. Thus, further studies with larger sample size are required to examine association in improvement of BMD with treatment type. Fourthly, the reported treatment persistence and compliance in our study could be influenced by their definitions and length of follow-up. For instance, a larger permissible gap period will inevitably result in higher estimated treatment persistence, while persistence tends to decrease with longer follow-up. Currently, there remains no standardized definition or methods for how medication persistence and compliance should be estimated [46]. It is also unknown whether patients who were prescribed with alendronate actually collected the medication or complied with the medication regimen at home. Lastly, although we had adjusted for several potential confounders in our regression analyses, it is likely that our results could still be influenced by residual confounding.

In conclusion, the 12-month analysis for this multicenter prospective study of postmenopausal women with osteoporosis showed that treatment persistence and compliance with denosumab were higher than weekly oral alendronate. Although treatment type was significantly associated with persistence and compliance, it was not found to be associated with improvement of BMD from baseline. Most patients in both treatment groups did not report any adverse events related to osteoporosis treatment. It is expected that our study findings will contribute to the understanding of real-world medication persistence and compliance among postmenopausal women with osteoporosis in the Asia–Pacific region. In addition, as our study was conducted during the COVID-19 pandemic, findings could also provide valuable insights into osteoporosis-related medication use during the pandemic.

## Supplementary Information

Below is the link to the electronic supplementary material.

Supplementary file1 (DOCX 206 KB)

## Data Availability

Qualified researchers may request data from Amgen clinical studies. Complete details are available at the following: https://wwwext.amgen.com/science/clinical-trials/clinical-data-transparency-practices/clinical-trial-data-sharing-request/
