# Supplementary material for: Real-world persistence and compliance of denosumab versus alendronate among postmenopausal women with osteoporosis in Asia–Pacific
Source: J Bone Miner Metab. 2025 Nov 19;44(1):69–83. doi: 10.1007/s00774-025-01663-2 (PMC12890978; doi:10.1007/s00774-025-01663-2)
Supplement: Supplementary file 1 — Supplementary file1 (DOCX 206 KB) [file 774_2025_1663_MOESM1_ESM.docx]

**Supplementary Materials****Real-world Persistence and Compliance of Denosumab Versus Alendronate Among Postmenopausal Women With Osteoporosis in Asia-Pacific**

Tang Ching Lau^1^, Dong-Gune Chang^2^, Chung-Hwan Chen^3^, Shuang Huang^4^, Edith MC Lau^5^, Sheung-Wai Law^6^, Young-Kyun Lee^7^, Cae Tolman^8^, Laura Canals^9^, See-Hwee Yeo^10^, Jing Yu^11^, Peter R. Ebeling^12^

^1^ Department of Medicine, Yong Loo Lin School of Medicine, National University of Singapore, Singapore, Singapore

^2^ Department of Orthopedic Surgery, Inje University Sanggye Paik Hospital, College of Medicine, Inje University, Seoul, South Korea

^3^ Department of Orthopedics and Orthopedic Research Center, Kaohsiung Municipal Ta-Tung Hospital and Kaohsiung Medical University Hospital, College of Medicine; Regeneration Medicine and Cell Therapy Research Center, Kaohsiung Medical University, Kaohsiung, Taiwan

^4^ Amgen Inc., Thousand Oaks, California, United States of America

^5^ Hong Kong Orthopaedic and Osteoporosis Center for Treatment and Research, Hong Kong

^6^ Department of Orthopaedics and Traumatology, The Chinese University of Hong Kong, Hong Kong SAR, China

^7^ Seoul National University Hospital, Seoul National University College of Medicine, Seoul, South Korea

^8^ Amgen Australia, Sydney, Australia

^9^ Department of Medicine, Amgen Europe, Switzerland

^10^ Real World Solutions, IQVIA Solutions Asia, Singapore, Singapore

^11^ Real World Solutions, IQVIA Solutions Asia, Beijing, China

^12^ Department of Medicine, School of Clinical Sciences at Monash Health, Monash University, Melbourne, Australia

**Corresponding authors**:

Laura Canals

Address: Amgen GmbH, Suurstoffi 22, 6343 Rotkreuz, Switzerland

Telephone: +41 79 9645

Email: [lcanals@amgen.com](mailto:ctolman@amgen.com)

Jing Yu

Address: IQVIA Data Science Advanced Analytics, The Exchange Twin Tower (West), B-12 Jianguomenwai Avenue, Chaoyang, Beijing, China

Telephone: +86 15219436846

Email: [jyu2@cn.imshealth.com](mailto:jyu2@cn.imshealth.com)

# Supplementary Figures and Tables


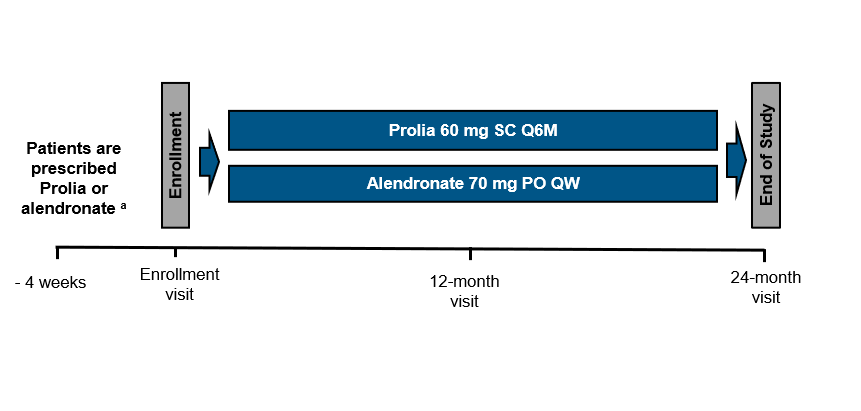


**Supplementary Figure 1:** Schematic diagram of study design.

Abbreviations: PO, orally; QW, weekly; Q6M, every 6 months; SC, subcutaneously.

^a^ At least 1 prescription for denosumab or alendronate should be provided to the patient before signing the informed consent form. A patient needs to be enrolled in the study within 4 weeks after receiving the first denosumab injection or first prescription of weekly alendronate for the current course of treatment.


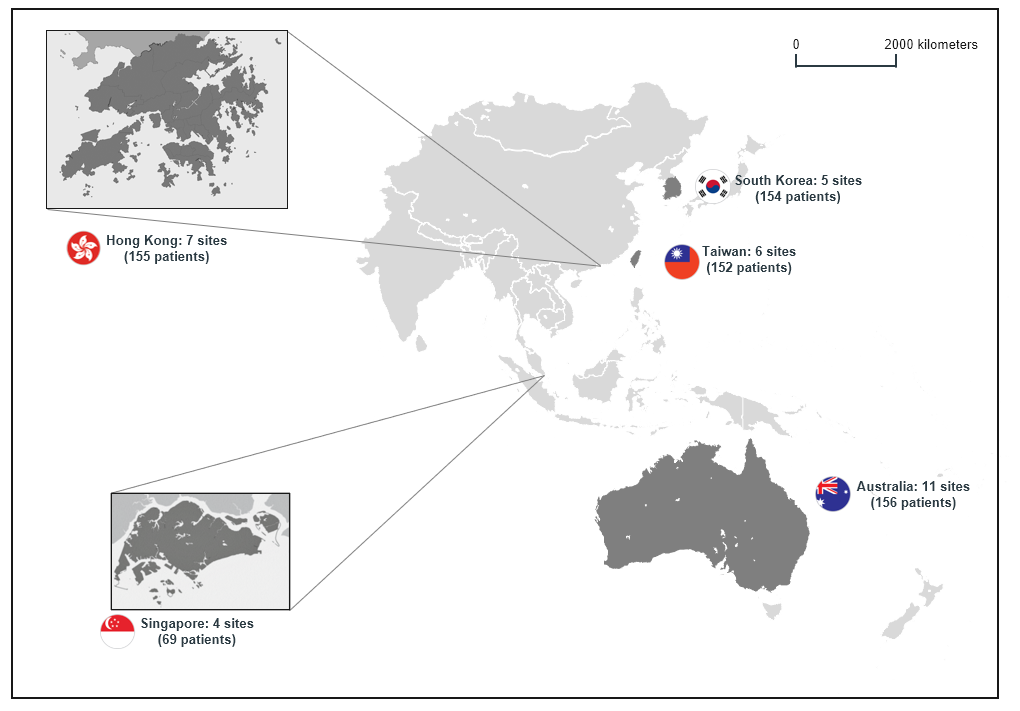


**Supplementary Figure 2.** Participating territories, sites, and enrolled patients

**Supplementary Table 1:** Subject accountability at 12-month visit.

| **Territory** | **Total** | **Denosumab** | **Alendronate** | ***P*-value ^a^** |
| --- | --- | --- | --- | --- |
| **All territories** | N = 686 | n = 350 | n = 336 |  |
| Completion of study | 580 (84.5%) | 301 (86.0%) | 279 (83.0%) | 0.283 |
| Withdrawal of study | 106 (15.5%) | 49 (14.0%) | 57 (17.0%) |  |
| **Australia** | n = 156 | n = 75 | n = 81 |  |
| Completion of study | 142 (91.0%) | 67 (89.3%) | 75 (92.6%) | 0.477 |
| Withdrawal of study | 14 (9.0%) | 8 (10.7%) | 6 (7.4%) |  |
| **Taiwan** | N = 152 | N = 77 | n = 75 |  |
| Completion of study | 109 (71.7%) | 57 (74.0%) | 52 (69.3%) | 0.521 |
| Withdrawal of study | 43 (28.3%) | 20 (26.0%) | 23 (30.7%) |  |
| **South Korea** | n = 154 | n= 77 | n = 77 |  |
| Completion of study | 124 (80.5%) | 65 (84.4%) | 59 (76.6%) | 0.222 |
| Withdrawal of study | 30 (19.5%) | 12 (15.6%) | 18 (23.4%) |  |
| **Hong Kong** | N = 155 | n = 76 | n = 79 |  |
| Completion of study | 141 (91.0%) | 70 (92.1%) | 71 (89.9%) | 0.628 |
| Withdrawal of study | 14 (9.0%) | 6 (7.9%) | 8 (10.1%) |  |
| **Singapore** | n = 69 | n = 45 | n = 24 |  |
| Completion of study | 64 (92.8%) | 42 (93.3%) | 22 (91.7%) | 0.799 ^b^ |
| Withdrawal of study | 5 (7.2%) | 3 (6.7%) | 2 (8.3%) |  |

^a^ Unless otherwise specified, *P*-values were generated by chi-square test for categorical variables.

^b^ Fisher's exact test was used to compare patients on denosumab and those on alendronate.

**Supplementary Table 2:** Adjusted odds ratios and 95% confidence intervals for osteoporosis treatment persistence at 12 months (sensitivity analysis).

| **Territory** | **Adjusted odds ratio^a^** | **95% CI** | ***P*-value** | **Reference group^b^** |
| --- | --- | --- | --- | --- |
| **All territories, (N = 580)** |  |  |  |  |
| Denosumab group | 3.05 | (2.03–4.60) | <.001* | alendronate |
| Age | 0.97 | (0.95–1.00) | 0.021* | - |
| History of fracture | 0.88 | (0.59–1.30) | 0.512 | no |
| Measurement of BMD | 0.94 | (0.60–1.48) | 0.783 | no |
| Prior treatment for osteoporosis | 1.48 | (0.99–2.19) | 0.055 | no |
| Prior treatment with oral glucocorticoids | 0.75 | (0.41–1.40) | 0.371 | no |
| Reimbursement status^c^ | 0.67 | (0.42–1.06) | 0.088 | no reimbursement |
| **Australia, (n = 142)** |  |  |  |  |
| Denosumab group | 19.42 | (7.13–52.89) | <.001* | alendronate |
| Age | 0.94 | (0.89–0.99) | 0.022* | - |
| History of fracture | 1.19 | (0.51–2.77) | 0.686 | no |
| Measurement of BMD | 1.39 | (0.38–5.12) | 0.622 | no |
| Prior treatment for osteoporosis | 0.50 | (0.19–1.30) | 0.155 | no |
| Prior treatment with oral glucocorticoids | 1.25 | (0.38–4.09) | 0.716 | no |
| **Taiwan, (n = 109)** |  |  |  |  |
| Denosumab group | 1.62 | (0.56–4.75) | 0.376 | alendronate |
| Age | 1.01 | (0.95–1.07) | 0.819 | - |
| History of fracture | 0.46 | (0.12–1.72) | 0.246 | no |
| Measurement of BMD | 3.40 | (0.80–14.45) | 0.097 | no |
| Prior treatment for osteoporosis | 2.95 | (0.63–13.69) | 0.168 | no |
| Prior treatment with oral glucocorticoids | 5.85 | (0.32–106.62) | 0.233 | no |
| **South Korea, (n = 124)** |  |  |  |  |
| Denosumab group | 1.81 | (0.60–5.45) | 0.291 | alendronate |
| Age | 0.94 | (0.88–1.00) | 0.048* | - |
| History of fracture | 0.48 | (0.16–1.45) | 0.195 | no |
| Measurement of BMD | 2.21 | (0.63–7.77) | 0.218 | no |
| Prior treatment for osteoporosis | 2.78 | (0.96–8.10) | 0.061 | no |
| Prior treatment with oral glucocorticoids | 0.46 | (0.13–1.64) | 0.229 | no |
| **Hong Kong, (n = 141)** |  |  |  |  |
| Denosumab group | 1.46 | (0.59–3.59) | 0.416 | alendronate |
| Age | 1.00 | (0.95–1.04) | 0.854 | - |
| History of fracture | 1.04 | (0.44–2.46) | 0.926 | no |
| Measurement of BMD | 1.72 | (0.55–5.34) | 0.348 | no |
| Prior treatment for osteoporosis | 2.18 | (0.82–5.83) | 0.119 | no |
| Prior treatment with oral glucocorticoids | 0.17 | (0.02–1.15) | 0.069 | no |
| Reimbursement status^c^ | 1.04 | (0.35–3.14) | 0.940 | no reimbursement |
| **Singapore, (n = 64)** |  |  |  |  |
| Denosumab group | 7.83 | (1.58–38.84) | 0.012* | alendronate |
| Age | 0.93 | (0.84–1.04) | 0.183 | - |
| History of fracture | 0.49 | (0.12–2.00) | 0.318 | no |
| Measurement of BMD | 1.44 | (0.29–7.06) | 0.653 | no |
| Prior treatment for osteoporosis | 0.96 | (0.23–3.98) | 0.949 | no |
| Prior treatment with oral glucocorticoids | 5.05 | (0.14–183.87) | 0.378 | no |

Abbreviations: BMD, bone mineral density; CI, confidence interval;

* *P* < 0.05

^a^ Adjusted odds ratios were generated using multivariable logistic regression models. Treatment persistence was used as the dependent variable, while adjusting for treatment group, age, history of fracture, measurement of BMD, prior treatment for osteoporosis, and prior treatment with oral glucocorticoids.

^b^ Treatment group is categorized as patients on denosumab and those on alendronate. History of fracture includes with fracture and without fracture. Measurement of BMD includes with BMD measurement and without. Prior treatment for osteoporosis includes using and not using osteoporosis drug before starting denosumab or alendronate treatment. Prior treatment with oral glucocorticoids includes using and not using oral glucocorticoids before starting denosumab or alendronate treatment.

^c^ Further adjusted for baseline reimbursement status if there are at least 10% subjects in both treatment groups for reimbursement categories.**Supplementary Table 3:** Changes in bone mineral density (T-score) at 12 months and their associations with osteoporosis treatment using multivariable linear regression.

| **Site** | **Denosumab (n = 301)** | | | **Alendronate (n = 279)** | | | **Regression coefficient (95% CI)^a^** |
| --- | --- | --- | --- | --- | --- | --- | --- |
|  | **Baseline mean (SD)** | **12-month mean (SD)** | **Change from baseline, mean (SD)** | **Baseline mean (SD)** | **12-month mean (SD)** | **Change from baseline, mean (SD)** |  |
| **Lumbar spine** |  |  |  |  |  |  |  |
| T-score | -2.4 (1.1) | -2.1 (1.0) | 0.3 (0.5) | -2.4 (0.9) | -2.2 (0.9) | 0.3 (0.6) | -0.05 (-0.20–0.10) |
| Percentage change in T-score from baseline (%) | - | - | 10.5 (27.3) | - | - | 13.0 (36.8) | -1.80 (-11.11–7.51) |
| **Total hip** |  |  |  |  |  |  |  |
| T-score | -2.0 (0.8) | -1.9 (0.8) | 0.2 (0.4) | -1.9 (0.9) | -1.8 (0.9) | 0.1 (0.3) | -0.06 (-0.15–0.03) |
| Percentage change in T-score from baseline (%) | - | - | 8.2 (15.7) | - | - | 4.0 (27.4) | -3.44 (-9.67–2.79) |
| **Femoral neck** |  |  |  |  |  |  |  |
| T-score | -2.4 (0.7) | -2.3 (0.7) | 0.1 (0.3) | -2.3 (0.9) | -2.2 (0.9) | 0.1 (0.5) | -0.01 (-0.12–0.11) |
| Percentage change in T-score from baseline (%) | - | - | 2.6 (22.9) | - | - | 5.7 (27.1) | 3.00 (-3.80–9.80) |

Abbreviations: CI, confidence interval; -, not applicable.

* *P* < 0.05.

^a^ Reference group for osteoporosis treatment comprised of patients on alendronate. Linear regression model was adjusted for age, baseline bone mineral density, history of fracture, prior treatment for osteoporosis, prior treatment with glucocorticoids, model of measuring device, history of falls or falls within last year, body mass index, smoking status, and ethnicity.

**Supplementary Table 4:** Association between variables and change in T-scores from baseline using multivariable linear regression.

| **Variables** | **Regression coefficient (95% CI)** | | |
| --- | --- | --- | --- |
|  | **Lumbar spine** | **Total hip** | **Femoral neck** |
| **Treatment** |  |  |  |
| Denosumab | -0.05 (-0.20–0.10) | -0.06 (-0.15–0.03) | -0.01 (-0.12–0.11) |
| Alendronate | Reference | Reference | Reference |
| **Age** | 0.01 (0.00–0.01) | 0.00 (-0.01–0.00) | 0.00 (-0.01–0.00) |
| **History of fracture** |  |  |  |
| Yes | 0.00 (-0.17–0.17) | -0.09 (-0.19–0.01) | -0.13 (-0.26–-0.01)* |
| No | Reference | Reference | Reference |
| **Baseline BMD** | -0.23 (-0.31–-0.16)* | -0.15 (-0.21–-0.10)* | -0.23 (-0.30–-0.16)* |
| **Prior treatment for osteoporosis** |  |  |  |
| Yes | -0.11 (-0.27–0.05) | -0.03 (-0.13–0.07) | 0.07 (-0.05–0.19) |
| No | Reference | Reference | Reference |
| **Prior treatment with glucocorticoids** |  |  |  |
| Yes | -0.02 (-0.23–0.19) | 0.07 (-0.07–0.21) | -0.06 (-0.23–0.11) |
| No | Reference | Reference | Reference |
| **Model of measuring device** |  |  |  |
| GE-Lunar | -0.50 (-0.96–-0.05)* | -0.16 (-0.50–0.18) | 0.06 (-0.33–0.44) |
| Hologic | -0.52 (-0.99–-0.06)* | -0.18 (-0.53–0.17) | -0.03 (-0.42–0.37) |
| Missing | Reference | Reference | Reference |
| **History of falls** |  |  |  |
| Yes | 0.23 (0.02–0.44)* | -0.01 (-0.14–0.12) | 0.00 (-0.16–0.15) |
| No | Reference | Reference | Reference |
| **Body Mass Index** | 0.01 (-0.01–0.03) | 0.02 (0.01–0.03)* | 0.01 (0.00–0.03) |
| **Smoking status** |  |  |  |
| Current smoker | 0.60 (-0.14–1.34) | 0.09 (-0.38–0.55) | -0.02 (-0.61–0.56) |
| Former smoker | 0.17 (-0.13–0.47) | 0.06 (-0.10–0.22) | -0.21 (-0.45–0.03) |
| Never | Reference | Reference | Reference |
| **Ethnicity** |  |  |  |
| Caucasian | -0.05 (-0.30–0.19) | 0.06 (-0.10–0.22) | 0.27 (0.07–0.46)* |
| Korean | -0.05 (-0.22–0.11) | 0.01 (-0.09–0.11) | -0.02 (-0.14–0.10) |
| Others^a^ | 0.10 (-0.54–0.73) | -0.05 (-0.51–0.41) | -0.01 (-0.59–0.57) |
| Chinese | Reference | Reference | Reference |

Abbreviations: BMD, bone mineral density; CI, confidence interval;

* *P* < 0.05

^a^ Malays and Indians were included in other ethnicities.

**Supplementary Table 5:** Association between variables and percentage change in T-scores from baseline using multivariable linear regression.

| **Variables** | **Regression coefficient (95% CI)** | | |
| --- | --- | --- | --- |
|  | **Lumbar spine** | **Total hip** | **Femoral neck** |
| **Treatment** |  |  |  |
| Denosumab | -1.80 (-11.11–7.51) | -3.44 (-9.67–2.79) | 3.00 (-3.80–9.80) |
| Alendronate | Reference | Reference | Reference |
| **Age** | 0.41 (-0.13–0.95) | -0.22 (-0.58–0.13) | -0.14 (-0.53–0.24) |
| **History of fracture** |  |  |  |
| Yes | -0.25 (-10.60–10.09) | -4.34 (-11.18–2.51) | -6.47 (-13.97–1.02) |
| No | Reference | Reference | Reference |
| **Baseline BMD** | -3.63 (-8.19–0.93) | -7.63 (-11.52–-3.73)* | -9.53 (-13.75–-5.31)* |
| **Prior treatment for osteoporosis** |  |  |  |
| Yes | -6.42 (-16.34–3.51) | 3.70 (-2.98–10.38) | 1.61 (-5.59–8.80) |
| No | Reference | Reference | Reference |
| **Prior treatment with glucocorticoids** |  |  |  |
| Yes | -8.04 (-21.26–5.18) | -0.65 (-10.29–8.99) | -1.56 (-11.73–8.61) |
| No | Reference | Reference | Reference |
| **Model of measuring device** |  |  |  |
| GE-Lunar | -10.48 (-38.60–17.64) | 0.05 (-23.07–23.18) | 12.56 (-10.35–35.46) |
| Hologic | -9.56 (-38.22–19.10) | -5.17 (-28.75–18.42) | 8.16 (-15.19–31.51) |
| Missing | Reference | Reference | Reference |
| **History of falls** |  |  |  |
| Yes | 13.55 (0.63–26.47)* | 3.52 (-5.13–12.18) | -0.41 (-9.81–8.99) |
| No | Reference | Reference | Reference |
| **Body Mass Index** | 0.64 (-0.52–1.80) | 1.43 (0.60–2.26)* | 0.46 (-0.39–1.31) |
| **Smoking status** |  |  |  |
| Current smoker | 37.44 (-8.28–83.15) | 6.89 (-24.44–38.23) | -3.65 (-38.28–30.97) |
| Former smoker | 9.24 (-9.15–27.62) | 4.52 (-8.64–17.68) | -11.47 (-25.53–2.59) |
| Never | Reference | Reference | Reference |
| **Ethnicity** |  |  |  |
| Caucasian | -2.53 (-17.56–12.50) | 2.63 (-8.11–13.37) | 16.81 (5.23–28.39)* |
| Korean | -2.55 (-12.77–7.67) | -3.92 (-10.60–2.77) | -3.66 (-10.94–3.62) |
| Others^a^ | -6.18 (-45.44–33.08) | -5.45 (-36.83–25.92) | -6.02 (-40.73–28.70) |
| Chinese | Reference | Reference | Reference |

Abbreviations: BMD, bone mineral density; CI, confidence interval;

* *P* < 0.05

^a^ Malays and Indians were included in other ethnicities.

**Supplementary Table 6:** Principal investigators and ethic approval authorities for each territory.

| **Site number** | **Territory** | **PI Name** | **Approval Code** | **Authority Name** |
| --- | --- | --- | --- | --- |
| 41 | Australia | Arya, Ktut | 2019-08-697-AA-PRE-1 | Bellberry Human Research Ethics Committees |
| 43 |  | Clifton-Bligh, Roderick | 2020 /STE01167 | Melbourne Health Human Research Ethics Committee |
| 35 |  | Cumming, Oscar | 2019-08-697 | Bellberry Human Research Ethics Committees |
| 39 |  | Gilfillan, Christopher | S20-014-56164 | Melbourne Health Human Research Ethics Committee |
| 29 |  | Inder, Warrick | HREC /56164 /MH-2019 | Metro South Health Service District Human Research Ethics Committee (RGO) |
| 45 |  | Inderjeeth, Charles | 2019-08-697 | Bellberry Human Research Ethics Committees |
| 30 |  | Seymour, Hannah | HREC /56164 /MH-2019 | Melbourne Health Human Research Ethics Committee |
| 40 |  | Stuckey, Bronwyn | 2019-08-697 | Bellberry Human Research Ethics Committees |
| 22 |  | Will, Robert | 2019-08-697 | Bellberry Human Research Ethics Committees |
| 42 |  | Wong, Peter | HREC /56164 /MH-2019 | Melbourne Health Human Research Ethics Committee |
| 23 |  | Yates, Christopher | HREC /56164 /MH-2019 | Melbourne Health Human Research Ethics Committee |
| 17 | Hong Kong | Cheung, Jason Pui Yin | UW19-545 | Institutional Review Board of the University of Hong Kong/Hospital Authority Hong Kong West Cluster |
| 14 |  | Ip, Tai Pang | UW19-749 | Institutional Review Board of the University of Hong Kong/Hospital Authority Hong Kong West Cluster |
| 18 |  | Kwok, Timothy Chi Yui | 2019.564 | Joint Chinese University of Hong Kong-New Territories East Cluster Clinical Research Ethics Committee |
| 12 |  | Lau, Edith | 2019 /03 | Hong Kong Doctors Union |
| 24 |  | Mok, Chi Chiu | NTWC /REC /19122 | New Territories West Cluster Ethics Committee |
| 36 |  | Wong, Ronald Man Yeung | 2019.579 | Joint Chinese University of Hong Kong-New Territories East Cluster Clinical Research Ethics Committee |
| 13 |  | Woo, Yu Cho | UW19-500 | Institutional Review Board of the University of Hong Kong/Hospital Authority Hong Kong West Cluster |
| 07 | South Korea | Chang, Dong-Gune | SGPAIK | IRB of Inje University Sanggye Paik Hospital |
| 15 |  | Koh, Jung-Min | 2019-04-007-001 | IRB of Asan Medical Center |
| 05 |  | Lee, Jae Hyup | 2019-0923 | IRB of Seoul Metropolitan Government Seoul National University Boramae Medical Center |
| 06 |  | Lee, Sang-Heon | 20190503 / 10 - 2019 - 42 / 061 | IRB of Konkuk University Medical Center |
| 20 |  | Rhee, Yumie | KUMC | IRB of Severance Hospital, Yonsei University Health System |
| 44 | Singapore | Chua, Ivan | 2019-06-035-004 | National healthcare group Domain specific review board. |
| 31 |  | Gani, Linsey | 4-2019-0803 | SingHealth Centralised Institutional Review Board |
| 27 |  | Mak, Anselm | 2019 /00858 | National Healthcare Group Domain Specific Review Board |
| 21 |  | Seng Bin, Ang | 2019 /2743 | SingHealth Centralised Institutional Review Board |
| 01 | Taiwan, Republic of China | Chen, Chung-Hwan | 2019 /00858 | Kaohsiung Medical University Chung-Ho Memorial Hospital, Institutional Review Board |
| 02 |  | Huang, Tsan-Wen | 2019 /2743 | Chang Gung Medical Foundation, Institutional Review Board |
| 04 |  | Kuo, Yi-Jie | KMUHIRB-E (I )-20190187 | Taipei Medical University, Joint Institutional Review Board |
| 08 |  | Lu, Ko-Hsiu | 201900706B0 | Chung Shan Medical University Hospital, Institutional Review Board |
| 10 |  | Niu, Chi-Chien | N201907008 | Chang Gung Medical Foundation, Institutional Review Board |
| 26 |  | Wu, Meng-Ting | CSMUH N0:CS2-19052 | Cheng Hsin General Hospital, Institutional Review Board |
